# Impact of media and antifoam selection on monoclonal antibody production and quality using a high throughput micro‐bioreactor system

**DOI:** 10.1002/btpr.2575

**Published:** 2017-11-16

**Authors:** Sai Rashmika Velugula‐Yellela, Abasha Williams, Nicholas Trunfio, Chih‐Jung Hsu, Brittany Chavez, Seongkyu Yoon, Cyrus Agarabi

**Affiliations:** ^1^ U.S. Food and Drug Administration, Center for Drug Evaluation and Research, Office of Product Quality, Office of Biotechnology Products, Division of Biotechnology Review and Research II Silver Spring MD; ^2^ Dept. of Chemical Engineering University of Massachusetts Lowell MA; ^3^ Present address: VPPL/VRC/NIAID/NIH Gaithersburg MD

**Keywords:** antifoam, media scouting, micro‐bioreactors, CHO cells, monoclonal antibodies

## Abstract

Monoclonal antibody production in commercial scale cell culture bioprocessing requires a thorough understanding of the engineering process and components used throughout manufacturing. It is important to identify high impact components early on during the lifecycle of a biotechnology‐derived product. While cell culture media selection is of obvious importance to the health and productivity of mammalian bioreactor operations, other components such as antifoam selection can also play an important role in bioreactor cell culture. Silicone polymer‐based antifoams were known to have negative impacts on cell health, production, and downstream filtration and purification operations. High throughput screening in micro‐scale bioreactors provides an efficient strategy to identify initial operating parameters. Here, we utilized a micro‐scale parallel bioreactor system to study an IgG1 producing CHO cell line, to screen Dynamis, ProCHO5, PowerCHO2, EX‐Cell Advanced, and OptiCHO media, and 204, C, EX‐Cell, SE‐15, and Y‐30 antifoams and their impacts on IgG1 production, cell growth, aggregation, and process control. This study found ProCHO5, EX‐Cell Advanced, and PowerCHO2 media supported strong cellular growth profiles, with an IVCD of 25‐35 × 10^6^ cells‐d/mL, while maintaining specific antibody production (Q_p_ > 2 pg/cell‐d) for our model cell line and a monomer percentage above 94%. Antifoams C, EX‐Cell, and SE‐15 were capable of providing adequate control of foaming while antifoam 204 and Y‐30 noticeably stunted cellular growth. This work highlights the utility of high throughput micro bioreactors and the importance of identifying both positive and negative impacts of media and antifoam selection on a model IgG1 producing CHO cell line. © 2017 The Authors Biotechnology Progress published by Wiley Periodicals, Inc. on behalf of American Institute of Chemical Engineers *Biotechnol. Prog.*, 34:262–270, 2018

## Introduction

Monoclonal antibodies (mAb) are an important class of biotechnology‐derived drug products with approved treatment indications ranging from autoimmune diseases, post‐transplantation complications, arthritis, and the treatment of various types of cancer.^1^ Nearly 70% of all recombinant protein production processes utilize Chinese Hamster Ovary (CHO) cells because of their ability to produce proteins with similar post‐translational modifications to that of an innate human protein, are readily adaptable to serum‐free media, and have demonstrated reliability as safe hosts.^2^

Due to the costs and complexities associated with the commercial production of mAbs, there is a strong demand to efficiently and economically deliver consistently high quality drug substance. While commercial yields for monoclonal antibodies may regularly yield ∼5 to 6 g/L, CHO cells can be genetically manipulated to further increase productivity.^3^ Increases in protein production have been attributed to bioprocess optimization due to advancements in media, cell culture conditions, and enhanced feeding strategies. Media composition is one of the most critical factors in mAb production as a balanced supply of micronutrients such as vitamins, amino acids, and trace metals, are required to support proper cell growth, and high quality protein production.^4^, ^5^, ^6^

Furthermore, choice of chemical antifoaming agents or “antifoams” is an important consideration that can impact the bioreactor process. Excessive foaming can result in damage to proteins from the bursting of bubbles, loss of control on system sterility from escaping foam, and pressure‐related damage to equipment from the blockage of exit filters.^7^, ^8^, ^9^ Antifoams are generally composed of solid hydrophobic particles, an oil, or a mixture of these components.^7^ Hydrophobic solid particles can disrupt foam through the bridge‐dewetting mechanism in which the hydrophobicity of the particle causes perforations on the surfaces of foam, causing it to rupture.^10^ Oil‐based antifoams similarly disrupt foams through the bridge‐dewetting mechanism, as well as the bridging‐stretching mechanism in which the oil forms bridges in the foam lamella that stretches and leads to the rupture of foam.^10^

Previous studies have shown that the addition of antifoam may have both positive and negative effects on cell growth, which may subsequently affect protein product yield.^11^, ^12^, ^13^ Silicone polymer‐based antifoams have been demonstrated to negatively impact cell growth in upstream bioprocessing and travel downstream where they can clog filters and create difficulties in purification steps.^14^ Furthermore, antifoams interact with cell membranes and can alter permeability resulting in leaking of cell contents and ultimately leading to cell death.^15^, ^16^ Massive cell death from antifoam toxicity to the cells can occur, but is considered a worst case scenario.^9^ With the wide variety of commercially available antifoams, it is important to select a formulation that fits the needs of the bioprocess.^9^

The objective of this research was to evaluate several combinations of commercially available media and antifoam for their impact on cell growth and specific productivity of an in‐house CHO‐DG44 cell line producing a model chimeric IgG1. We used an automated microbioreactor ambr®15 (Sartorius, Hertfordshire, UK), consisting of 48 parallel microbioreactors that have been shown to be comparable to classical stirred tank reactors in scale up studies.^17^ We evaluated five chemically defined CHO media and five antifoams to determine impacts on cell growth, and antibody production. Based on preliminary batches, the media and antifoam selections were narrowed down to three antifoams and three media, keeping OptiCHO as a reference/baseline media. While optimization of media strategies requires a deep understanding of the process and feeding needs, it is extremely valuable to eliminate poor performers and detrimental antifoams that may have advertised applications for a broad scope of CHO cell lines (such as DG‐44 or CHO suspension), and quickly narrow down the media candidates for our specific model cell culture system.

## Materials and Methods

### Cell culture reagents

Chemically defined (CD) OptiCHO medium (Thermo Fisher Scientific, Waltham, MA) was the original media in which the cell line was propagated. The cells were adapted to four other commercially available media, including CD Dynamis (Thermo Fisher Scientific, Carlsbad, CA), protein‐free ProCHO 5 (Lonza, Walkersville, MD), CD PowerCHO 2 (Lonza, Walkersville, MD), and CD EX‐Cell Advanced (SAFC, Lenexa, KS). All media studied were supplemented with 8 mM L‐glutamine (Corning, Manassas, VA) and 1X Penicillin/Streptomycin (Corning, Oneonta, NY). Initial glucose concentration in each media varied, based on the manufacturer's proprietary formulation, and ranged between 5 and 8 g/L. ProCHO 5 and EX‐Cell Advanced had the highest glucose concentration, while OptiCHO had the lowest glucose concentration. Five commercially available antifoams (Sigma Life Sciences, St. Louis, MO) were studied: Antifoam C, Antifoam EX‐Cell, Antifoam 204, Antifoam SE‐15, and Antifoam Y‐30. Cell culture pH was controlled using 1M NaOH (Thermo Fisher Scientific, Fair Lawn, NJ).

### Seed train expansion

An in‐house, chimeric IgG1 producing CHO DG44 cell line was cultured in the five test media. Shake flask cultures (30 mL) were maintained in an incubator controlled at 37°C and 8% CO_2_, and were agitated at a speed of 130 rpm. On day three, cells in each media were sub‐cultured into a 125‐mL single‐use spinner‐flask (Corning) containing fresh media. Spinner cultures were incubated at the same conditions as the shaker flasks and stirred at a speed of 70 rpm. Additional fresh media was added to the spinner cultures on day three (one day prior to inoculum preparations) to maintain cell viability above 90% with a maximum volume of 125 mL.

### Microbioreactor system batch process

Batch cultures for all experiments were maintained in a 15 mL advanced microscale bioreactor system consisting of 48 disposable vessels (sparged or non‐sparged) within 4 workstations. Process set points in each vessel were identical: dissolved oxygen (DO) 50%, pH 7.1 ± 0.05, temperature 37°C, and agitation speed 1000 rpm. A liquid handler provided automated media charging, inoculation, sampling, and additions of antifoam and NaOH. After inoculating at either 0.5 × 10^6^ cells/mL or 1 × 10^6^ cells/mL, the culture volume was maintained above 10 mL. Viable cell density and cell viability were measured daily using an automated and integrated Vi‐Cell XR cell viability analyzer (Beckman Coulter, Brea, CA). Daily samples were taken from each vessel for nutrient analysis (glucose, glutamine, and lactate) with a BioProfile FLEX analyzer (Nova Biomedical, Waltham, MA). Base (1 M NaOH) was added on an as needed basis for each individual sparged vessel throughout culture duration (8–9 days).

Nutrient and metabolic by‐product consumption and production rates were calculated for the linear region of the exponential growth phase only; this region was determined by visual assessment of the cell density's temporal evolution by a cell culture expert. A linear regression was then performed on all measurements that fall within this region, and the slope of the resulting regression line is taken to be the nutrient consumption rate, or the by‐product accumulation rate. Supplementary Figure S3 shows an example of this procedure.

**Figure 1:**
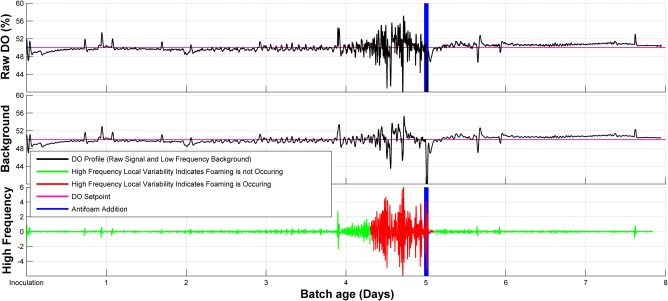
Assessing local variability in the high frequency components of the DO signal. (Top) The raw DO signal. (Middle) The low frequency background signal. This was acquired from a third‐order Savitzky‐Golay smoothing filter with a window size of 31 data points. (Bottom) The high frequency components of the DO signal.

Antifoam was added to the vessels based on visual observation of foam in culture vessels alone during the media scouting run, although it was noticed that dissolved oxygen measurements, shown in the top of Figure 1 for a single vessel, could be used as an indirect way to monitor when foaming occurred. This was accomplished by using a third‐order Savitzky‐Golay smoothing filter, with a window size of 31 data points, to obtain the low frequency background signal shown in the middle of Figure 1.^18^ The calculation of the low frequency background values, Y_j_*, from the raw measurements, *Y_j_*, is given by Eq. (1), where *m* is the window size and *C_i_* are convolution coefficients that depend on the order and window size, and it was evaluated using MATLAB. The background was subtracted from the raw signal, as given by Eq. (2), to obtain the high frequency signal, *Y* ^HF^, shown in the bottom of Figure 1. It was seen that when the local variability, calculated from Eq. (3) with a window size, *m*, of 151 data points, exceeded 250% of the global variability, calculated from Eq. (3) for all prior data points, then foaming was occurring, as indicated by the red portion of the signal in the bottom of Figure 1. In the antifoam scouting and confirmation runs, foaming was assessed from the high frequency, local variability in the DO measurement, and was confirmed visually for vessels in the front row of the AMBR system.

(1) $$Y_{j}^{*}=\frac{1}{m}\sum_{i=-\frac{m-1}{2}}^{\frac{m-1}{2}}C_{i}Y_{j+i}$$

(2) $$Y_{j}^{HF}=Y_{j}-Y_{j}^{*}$$

(3) $$\sigma_{j}^{2}=\frac{1}{m-1}\sum_{i=0}^{m-1}{\left(Y_{j-i}^{HF}-\mu_{j}\right)}^{2}$$

### Antifoam preparations

Antifoam 204 is an organic, non‐silicone polypropylene‐based polyether dispersion while Antifoam C, SE‐15, and Y‐30 are silicon emulsions. Antifoam EX‐Cell is a simethicone emulsion. For antifoams C, SE‐15, 204, and Y‐30 a 3% (v/v) working solution was prepared using ultra‐pure deionized water. The solutions were then autoclaved to maintain sterility, except Antifoam Ex‐Cell which is a pre‐sterilized 1.3% ready to use solution. All antifoams were prepared and stored in accordance with manufacturer specifications. For the media screening run, additions of 50 µL of 3% antifoam C solution (30ppm) was sufficient to mitigate foaming. For the subsequent antifoam screening, comparisons were made using the same initial 30 parts per million (ppm) of active ingredient in to the microbioreactor. Subsequent additions of antifoam were kept at 30 ppm per addition.

### Antibody titer and quality analysis

Titer and product quality assays were run on the last day of culture. Cell‐free harvested cell culture fluid (HCCF) was sterile filtered using 0.22 µm syringe filters (Millipore). Sterile HCCF was then evaluated for antibody concentration using an Octet Red 96 (Pall Life Sciences, Port Washington, NY) with Protein A biosensors and compared to a previously generated standard curve.^19^ Specific Productivity (*Q*_p_) was calculated by dividing the IgG1 titer measurement from the Octet Red on the last day of the culture and dividing by the IVCD of the culture, calculated over the entire time of culture, normalizing protein production and facilitating comparison between fast and slow growing cell cultures.

The sterile HCCF was purified using an AKTA Avant Protein Purification System (GE Healthcare Life Sciences, Pittsburg, PA). Prosep® vA Ultra resin (Millipore, Billerica, MA) was packed to 10 cm into an Omnifit (Diba Fluid Intelligence, Danbury, CT) glass housing with 0.66 m diameter. Equilibration buffer used was 25 mM Tris (Diluted from 1 M Tris buffer at pH 7.5(Alfa Aesar, Ward Hill, MA)) with 100 mM NaCl (Thermo Fisher Scientific, Fair Lawn, NJ) at a pH of 7.5. For wash step 25 mM Tris with 1 M NaCl at pH 7.5 was used and for elution 0.1 M glacial acetic acid was used. The protein concentrations of the eluted fractions were then measured using the Nanodrop^TM^ 2000/200c spectrophotometer (Thermo Fisher Scientific, Madison, WI).

Protein aggregation was analyzed using size‐exclusion chromatography. An Agilent SEC‐3 3 µm, 300Å 7.8 × 300 mm SEC column was used as the stationary phase (Agilent Technologies, Santa Clara, CA) and the mobile phase was 1X PBS (Quality Biological, Gaithersburg, MD) at a flow rate of 0.5 mL/min. The column was maintained at room temperature with an injection volume of 20 µL. The protein signal was measured by a UV detector at 280 nm.^20^

## Results

Robust and efficient media performance is a critical component for cell culture bioreactor production of biotechnology products. The effects of cell culture media and antifoam combinations on specific productivity (*Q*_p_) of the cells can be system specific. In our case, we investigated our model system to reveal what is optimal and which combinations of media and antifoam can be detrimental to cell culture health. The results were further verified by a subsequent confirmation experiment.

### Media scouting analysis

**Figure 2:**
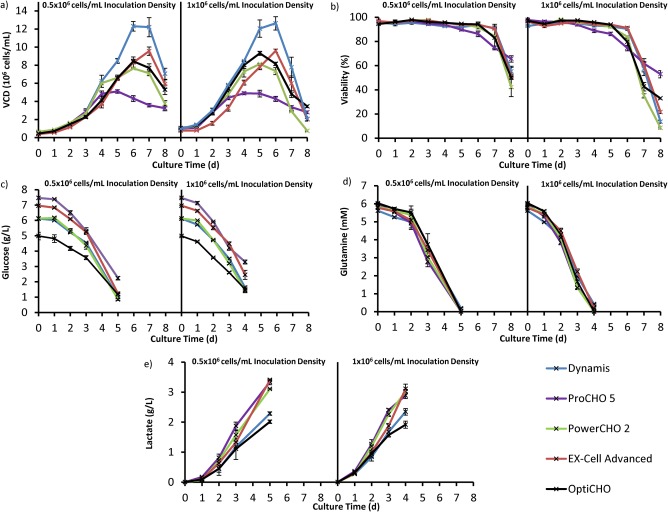
Cell growth characteristics for media scouting analysis. For both panels, the lower inoculation density graph is on the left, while the higher inoculation density is shown on the right. (a) Viable cell density averaged across same media conditions for two different inoculation densities. The plots indicate relative growth performance with Dynamis having the highest growth rate and ProCHO5 having the lowest. (b) Cell viability averaged across the same media conditions. (c)‐(e) Nutrient and by‐product profiles averaged across same media conditions at two different inoculation densities. Error bars correspond to ±standard deviation.

Five media were investigated for their potential to improve cell growth, antibody production, and percentage monomer in our system. CHO cells producing a chimeric, monoclonal IgG1 were cultured in batch mode using an ambr15 microbioreactor system and were inoculated at either 0.5 × 10^6^ or 1 × 10^6^ cells/mL in Dynamis, ProCHO 5, PowerCHO 2, EX‐Cell Advanced, or OptiCHO media. Growth and viability profiles for cells cultured in each of these media are shown in Figure 2.

While all five media are advertised as appropriate CHO expression media specifically for CHO DG44 or general CHO suspension culture media (ProCHO5), there are clear differences in the cellular growth profiles in our model system. As shown in Figure 2, cultures grown in Dynamis demonstrated the highest growth rate followed by EX‐Cell Advanced, PowerCHO2, and OptiCHO for both inoculation densities. ProCHO5 had the lowest growth rate and an extended stationary phase. The growth profile shifted back by 1 day for the higher inoculation density cultures. Within each media, it appears that the exponential phase starts 1 day earlier for the higher inoculation density.

**Figure 3:**
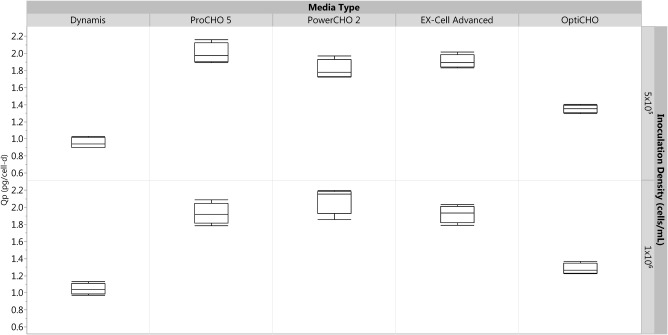
Box‐plot for assessing relative specific productivity between various media types for two different inoculation densities on day 8 of the culture. ProCHO5, EX‐Cell Advanced, and PowerCHO2 media all have relatively higher specific productivity when compared to the OptiCHO baseline. All conditions were run in quadruplicate.

As cellular growth rate may not always be a predictor of antibody titer, specific productivity of the cells in each media was also considered. From Figure 3, we can observe that Dynamis has the lowest specific productivity relative to the other media. ProCHO5, EX‐Cell Advanced, and PowerCHO2 had similar specific productivity, and all performed better than the OptiCHO baseline. The assessment for Figure 3 was performed relative to the OptiCHO media baseline within the batch process for both inoculation densities. There was no considerable difference in specific productivity between the two different inoculation densities; however, the variance was higher at lower inoculation densities for each media.

### Antifoam scouting analysis

**Figure 4:**
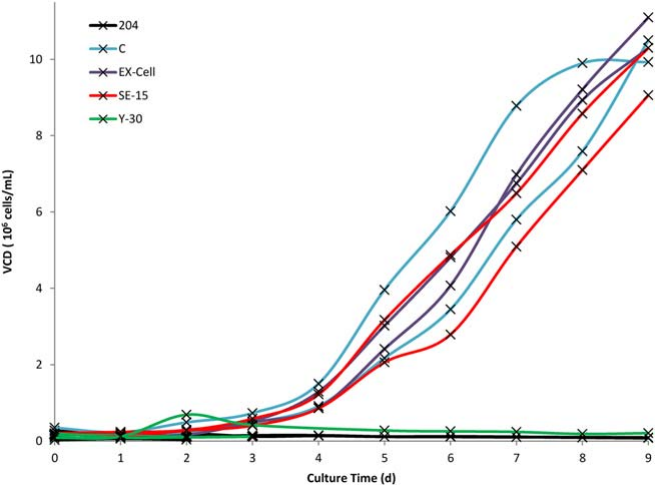
Representative viable cell density of cultures from five antifoams in EX‐Cell Advanced Media. Y‐30 and 204 antifoams demonstrably inhibited cell growth. The remaining three antifoams were comparable. The experiment was run in triplicates.

To examine the impact of antifoam additions on cell growth and antibody production yield, five antifoams were investigated. Antifoam 204 is organic non‐silicone dispersion while antifoams C, SE‐15, and Y‐30 are silicone‐based emulsions. EX‐Cell antifoam is a simethicone emulsion. This run was used to eliminate antifoams that are detrimental or toxic to cell growth. Figure 4 shows representative cell growth profiles for each antifoam type in EX‐Cell media, which mirrors the trends as observed in all other media (Supplementary Figure S1). As shown in Figure 4, antifoams 204 and Y‐30 (with the exception of combination with PowerCHO2) proved to be toxic to the cells and were excluded from further study. Apart from cell growth, we looked at specific productivity (Supplementary Table S2) of cells across all three media for EX‐Cell, C, and SE‐15 antifoams and they were found to be between 1.5 and 2.3 pg/cell‐d. Except for PowerCHO2, antifoam C had relatively high specific productivity for Ex‐Cell Advanced and ProCHO5 media. The SEC data yielded percent monomer above 94% across all media.

### Confirmation study

**Figure 5:**
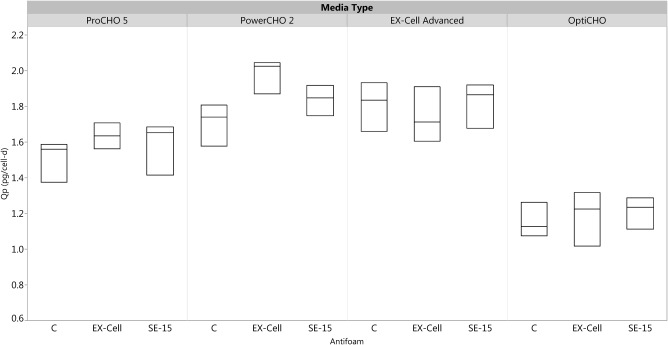
Box‐plot of the specific productivity of the cells in all possible combinations of three media types, EX‐Cell Advanced, ProCHO5, and PowerCHO2 and three antifoam types, C, EX‐Cell, and SE‐15.

To conclude that the results from the media scouting analysis and the antifoam scouting analysis were reproducible, the experiment was run again with the three media and the three antifoams that showed good specific productivity. OptiCHO was used as the baseline medium for comparison. The specific productivity for the validation batch is as shown in Figure 5.

As shown in Figure 5, the specific productivity of cells cultured in ProCHO5, EX‐Cell Advanced, and PowerCHO2 was higher than those of cells cultured in OptiCHO, confirming the media scouting analysis results that these media were an improvement compared to the baseline media. These results were consistent across all three antifoams. For each of the three media types, the specific productivity was the similar for all three antifoams, confirming the trends from the antifoam scouting analysis.

## Discussion

### Media scouting analysis

Changes to the quality attributes in final antibody product can often be traced back to changes in media and/or process parameters. Therefore, media evaluation in the early stages of process development can yield critical insights in to cell culture performance and product quality serving as a starting point to more extensive development studies.

In our media scouting analysis we observed that the cells cultured at an inoculation density of 1 × 10^6^ cells/mL provided marginally higher titers than those cultured at the lower density of 0.5 × 10^6^ cells/mL, with relative ratios between the same media remaining consistent in comparison to OptiCHO medium. The cells cultured at an inoculation density of 1 × 10^6^ cells/mL also had a shorter culture time and provided more consistent growth profile across all replicates in each media. Dynamis media provided the highest cell growth, having the largest average IVCD of 52 ×10^6^ cells‐d/mL. Despite the more robust cell growth profiles in Dynamis media, cells did not produce more IgG in this media. PowerCHO 2, EX‐Cell Advanced, and OptiCHO provided slightly lower growth performances with mean IVCDs of 34–41 × 10^6^ cells‐d/mL (Supplementary Table S1). The growth profile of cells cultured in ProCHO 5 media is unique when compared to the other media types studied. These cells reached a much lower IVCD at 27 × 10^6^ cells‐d/mL, and had a more gradual death phase when compared to the other media, despite a higher glucose concentration (Supplementary Table S1).

Generally, robust cell growth in terms of high cell densities and low lactate accumulation has been found to correlate with high titer.^21^ However, this did not hold true for the CHO cell line used in this study. While Dynamis media provided the highest cell densities and lowest lactate conditions, the IgG titers were lower than those of cells cultured in the baseline media, OptiCHO. Cells cultured in PowerCHO 2 and EX‐Cell Advanced produced the highest titer of antibody with nearly 27% more production than OptiCHO cultures. The higher titer observed in PowerCHO 2 and EX‐Cell Advanced also correlated with a higher specific productivity (*Q*_p_) of ∼2 pg/cell‐d. Cells cultured in ProCHO5 showed the lowest overall titer but had a specific productivity comparable to PowerCHO2 and EX‐Cell Advanced with a *Q*_p_ of ∼2 pg/cell‐d. *Q*_p_ for cells in Dynamis and OptiCHO were lower at 1‐1.3 pg/cell‐d indicating a higher per cell IgG production in ProCHO5.

As shown in Table 1, OptiCHO has the lowest glucose consumption rates while Ex‐Cell Advanced had the highest glucose consumption rates. OptiCHO media had the lowest lactate production rate while Ex‐Cell advanced had the highest lactate production rate. An unbalanced supply of nutrients in media has been found to correlate with high lactate and ammonium build‐up which can inhibit cell growth and decreases protein productivity.^22^

**Table 1:** Specific Productivity, Nutrient Consumption, and Production Rates of Cultures in All Five Media, at Two Different Inoculation Densities. Data Represented as Mean; *n* = 4

Media	*Q*_p_ (pg/cell‐d)	Consumption Rates		Production Rates
		Glucose (g/L‐day)	Glutamine (mMol/L‐day)	Lactate (g/L‐day)
*0.5 × 10^*6*^ cells/mL inoculation density*				
Dynamis	0.95	1.411 ± 0.087	1.590 ± 0.232	0.580 ± 0.061
ProCHO 5	2.00	1.023 ± 0.086	1.349 ± 0.100	0.841 ± 0.084
PowerCHO 2	1.81	1.471 ± 0.055	1.673 ± 0.211	0.758 ± 0.041
EX‐Cell Advanced	1.91	1.620 ± 0.053	1.630 ± 0.141	0.907 ± 0.047
OptiCHO	1.35	0.984 ± 0.02	1.772 ± 0.151	0.494 ± 0.046
*1 × 10^*6*^ cells/mL inoculation density*				
Dynamis	1.04	1.453 ± 0.169	1.687 ± 0.119	0.725 ± 0.066
ProCHO 5	1.93	1.411 ± 0.071	1.902 ± 0.112	0.970 ± 0.047
PowerCHO 2	2.09	1.576 ± 0.084	1.976 ± 0.043	0.967 ± 0.039
EX‐Cell Advanced	1.92	1.668 ± 0.225	2.357 ± 0.182	1.147 ± 0.194
OptiCHO	1.28	1.063 ± 0.123	1.992 ± 0.079	0.563 ± 0.073

The formation of protein aggregates and fragments during mAb production is an early sign of protein instability, which can lead to an immunogenic response in patients.^23^ Protein aggregation and fragmentation can be affected by pH, temperature, and dissolved oxygen within the bioreactor as well as media components.^24^, ^25^, ^26^ The quality of the resulting mAb was evaluated for each media type by size‐exclusion chromatography to determine the percentage of monomer and high‐molecular weight, or low‐molecular weight forms. Previous reports of mAb quality during the cell culture process have shown aggregation levels as high as 30%.^27^ The percentage monomer in these studies was consistent across all conditions to be >94% (Supplementary Figure S2).

Due to the high titer and productivity of cells cultured in PowerCHO 2 and EX‐Cell Advanced, these media were evaluated further. ProCHO 5 was selected for additional evaluation because of its unique and more gradual death phase as well as its high *Q*_p_. Previous research has found that optimal cell culture performance in batch mode may not translate to desirable fed‐batch culture performance,^21^ and has indicated that protein expression is often enhanced during stationary phase where reduced growth rates may lead to lower metabolic waste production and increased protein production.^28^ Thus, it is possible that ProCHO 5 media may perform better in future studies in which cells will be cultured in fed‐batch or perfusion modes, despite these initial findings of lower cell growth and higher lactate production rates.

### Antifoam scouting analysis

Based on the initial media screening run ProCHO5, PowerCHO2, and EX‐Cell Advanced were found to be better than Dynamis and OptiCHO in terms of specific productivity. These three media were used to evaluate various antifoams. Conditions that affect foaming in bioreactors include culture medium, cellular metabolites, cell growth, and gas introduction. Antifoam C used during initial media screening was successful in de‐foaming the culture. However, the addition of silicone antifoam is known to result in the reduction of both hydrodynamic and mass transfer characteristics, as well as alteration of cell growth rate, cell morphology and product production, allowing for further optimization of the cell culture process via antifoam selection.^29^ While the effects on mass transfer may decrease over the duration of the culture for silicone‐based de‐foamers, non‐silicone antifoams do not have these same affects.^7^ However, the effect of antifoam on cell culture growth and productivity has not been widely investigated. Therefore, we selected five commercially available antifoams from a vast array of silicone and non‐silicone antifoams.

Each combination of media and antifoam type was run in triplicate, with some replicates in the front row and some replicates in the back row in the ambr15 system. However, only the front row of vessels within each culture station could be seen, which allowed their foam level to be assessed visually; the vessels in the back were not visible and could not be monitored in the same manner. It was assumed that the vessels in back would be foaming around the same time as the replicate vessels in front, so antifoam was added to the vessels at the same time. Their DO profiles were used as a basis to justify this assumption.

**Figure 6:**
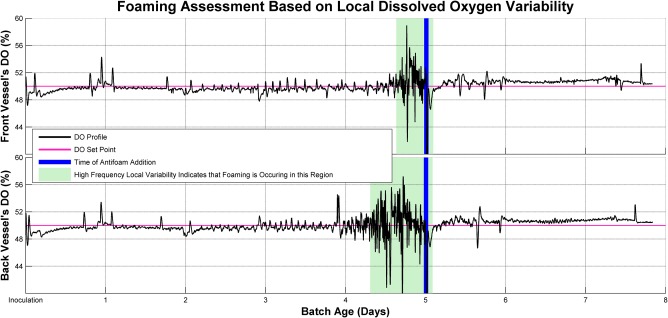
Foaming assessment based on local dissolved oxygen variability. High frequency local variability indicates the occurrence of foam in culture.

It was observed that as foaming increases, the high frequency local variability in the DO measurements grew larger. This is may have been due to a decrease in the volumetric oxygen transfer coefficient in the presence of air bubbles on the fluid surface.^30^, ^31^ After antifoam addition, the high frequency local variability in the DO measurements returned to the levels seen before foaming began. As most small scale reactors do not have foam sensors, the variability in DO profile may serve as a potential tool in developing foam control strategy. Figure 6 shows the DO profile for two vessels containing EX‐Cell Advanced media with SE‐15 antifoam, which was selected as a representative of all the vessels for illustration purposes. It can be seen that the high frequency local variability increased around the same time for both vessels, albeit slightly earlier for the vessel in back. It can also be seen that after the antifoam addition, the high frequency local variability decreased to normal vessels shortly thereafter. This served to confirm that the foam level was being controlled in the rear vessels that could not be inspected visually.

The total volume of antifoam added, cell growth and specific productivity were considered in determining sufficient antifoam conditions. In the initial antifoam batch, we found that antifoam 204 and Y‐30 were toxic to the cells and inhibited cell growth. Only when PowerCHO 2 media containing Y‐30 antifoam was used did the cells survive, and their performance with respect to IVCD and specific productivity was less than those of antifoams C, EX‐Cell, and SE‐15. In each media the IVCD of antifoam C, EX‐Cell, and SE‐15 were 15–35 × 10^6^ cells‐d/mL. Antifoam C had a higher titer when compared to antifoams EX‐Cell and SE‐15 in ProCHO 5 and PowerCHO2 but EX‐Cell antifoam performed better in EX‐Cell media in terms of titer (Supplementary Table S2). Except in PowerCHO2 media, antifoam C has relatively high specific productivity for the other two media tested (Supplementary Table S2).

### Confirmation study

Three media, including ProCHO5, PowerCHO2, and EX‐Cell Advanced, were compared to baseline OptiCHO media in combination with antifoams C, EX‐Cell, and SE‐15. We were able to confirm our results from media scouting analysis and the antifoam scouting analysis. It was observed that ProCHO5, PowerCHO2, and EX‐Cell Advanced medium still performed better than the baseline OptiCHO medium for cell growth and cell specific productivity of antibody, irrespective of the antifoam used. It was also seen that antifoam did not negatively impact the cell specific productivity in all the three media analyzed. Antifoams C, EX‐Cell, and SE‐15 performed similarly with respect to cell growth and specific productivity.

## Conclusion

In summary, this work demonstrated the importance of system‐specific evaluations of multiple media and antifoam types and their potential impact on high quality antibody production in cell culture. ProCHO5, EX‐Cell Advanced, and PowerCHO2 media were found to perform better for our model cell line, but media and antifoam selections should be considered on an individual product and process basis. In this evaluation of antifoams, some more clearly demonstrated cell toxicity, with Antifoam C, EX‐Cell, and SE‐15 performing most effectively to control foaming while minimizing the total amount of silicone antifoam used. These findings are meant to serve as a case study for our specific monoclonal antibody producing system and support input settings for future experiments to optimize media with proper supplementation (e.g. amino acids, vitamins, and trace metals to the media), while other cell lines, configurations, and manufacturing aims may require unique optimization strategies.

## Supporting information

Supplementary Figure 1 Black and White

Supplementary Figure 1 Color

Supplementary Figure 2

Supplementary Figure 3

Supplementary Table 1

Supplementary Table 2
