# Supplementary figures and images for: Impact of media and antifoam selection on monoclonal antibody production and quality using a high throughput micro‐bioreactor system
Source: Biotechnol Prog. 2017 Nov 16;34(1):262–70. doi: 10.1002/btpr.2575 (PMC5821576; doi:10.1002/btpr.2575)

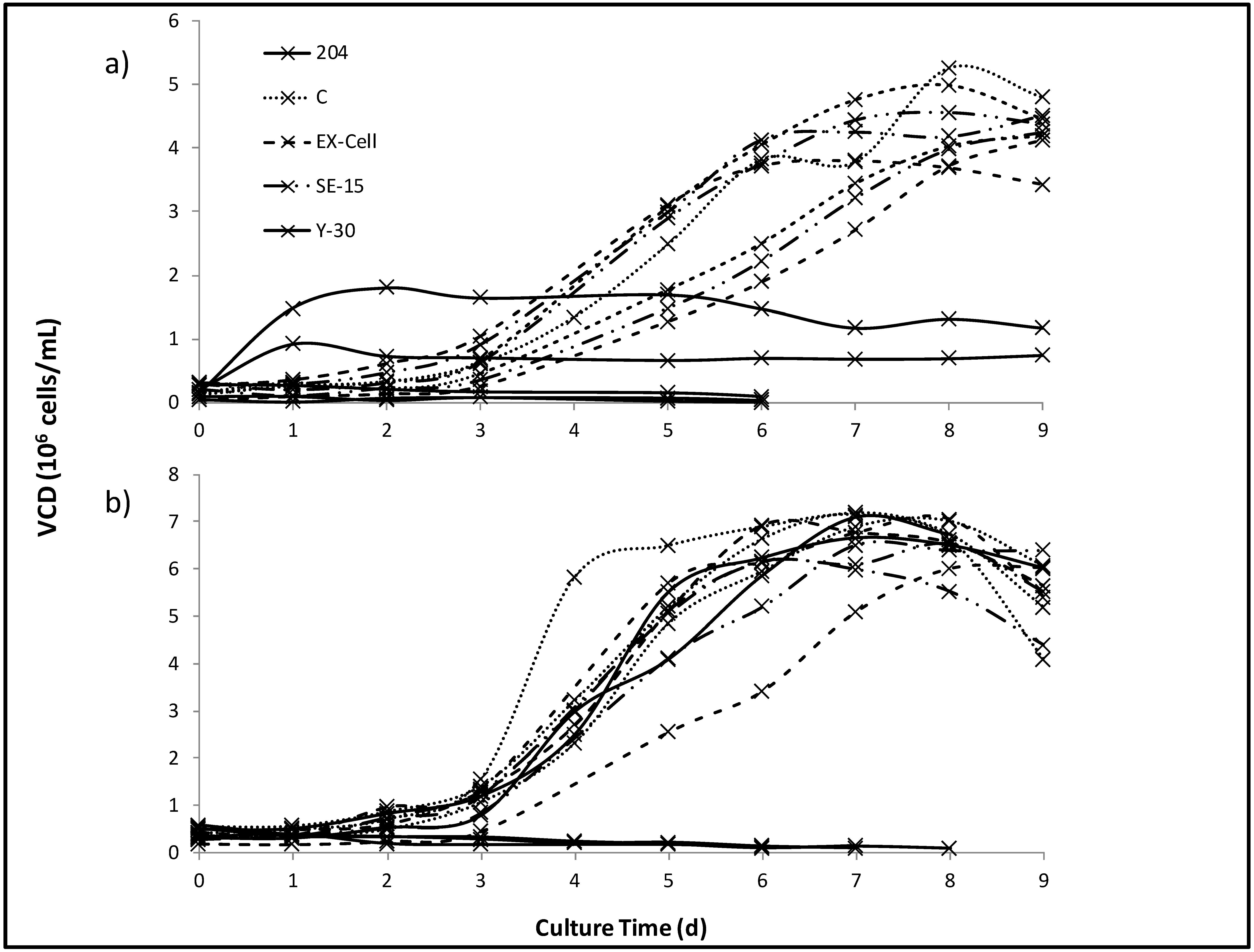

Supplement: Supplementary file 1 — Supplementary Figure 1 Black and White [file BTPR-34-262-s001.tiff]

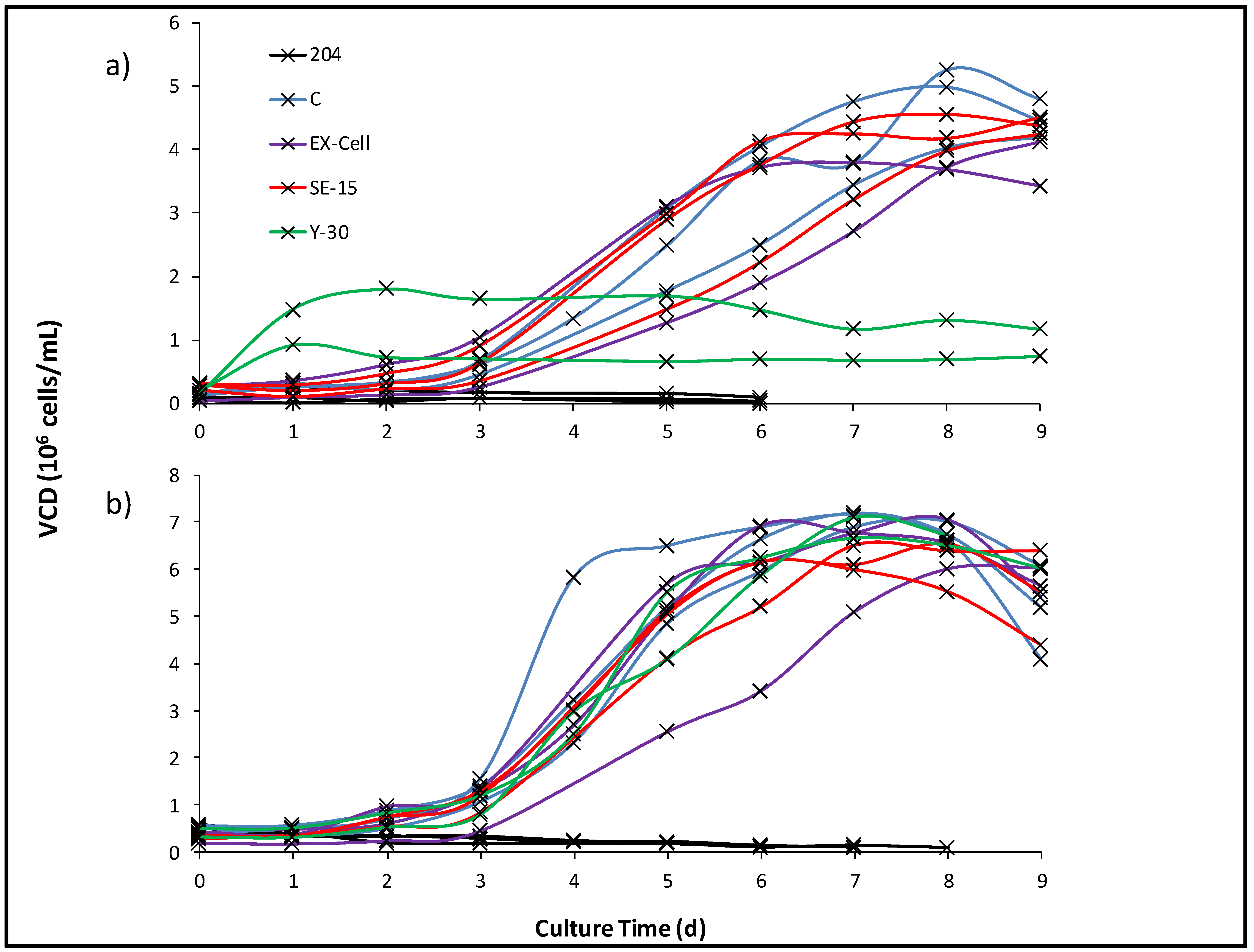

Supplement: Supplementary file 2 — Supplementary Figure 1 Color [file BTPR-34-262-s002.tiff]

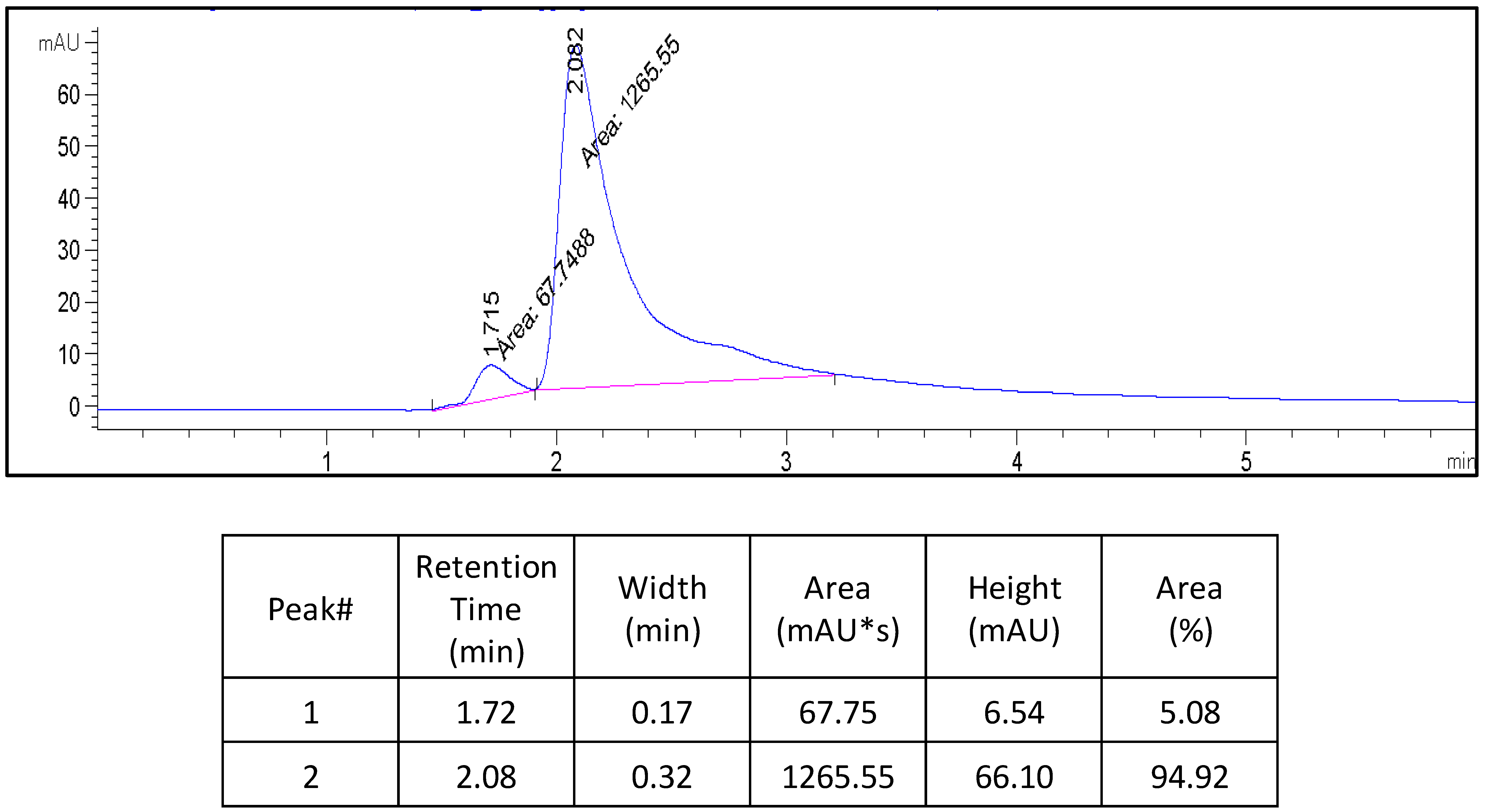

Supplement: Supplementary file 3 — Supplementary Figure 2 [file BTPR-34-262-s003.tiff]

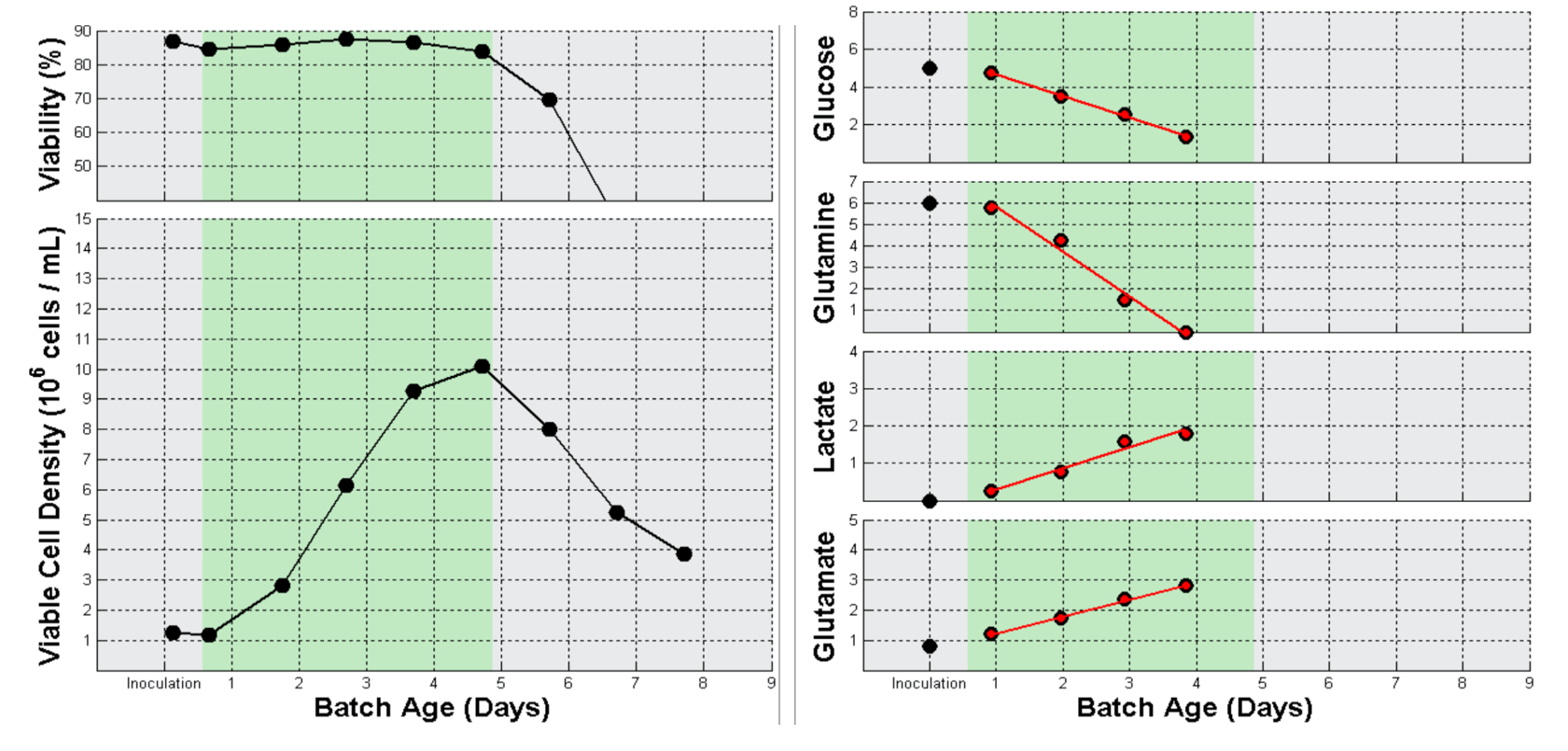

Supplement: Supplementary file 4 — Supplementary Figure 3 [file BTPR-34-262-s004.tif]
