# Supplementary material for: Impact of media and antifoam selection on monoclonal antibody production and quality using a high throughput micro‐bioreactor system
Source: Biotechnol Prog. 2017 Nov 16;34(1):262–70. doi: 10.1002/btpr.2575 (PMC5821576; doi:10.1002/btpr.2575)
Supplement: Supplementary file 5 — Supplementary Table 1 [file BTPR-34-262-s005.docx]

| **Media** | **Mean IVCD**  **( 10^6^ cells-d/mL)** | **Titer**  **(mg/L)** | **Average**  **% Monomer** |
| --- | --- | --- | --- |
| **0.5×10^6^ cells/mL Inoculation Density** | | | |
| **Dynamis** | 47.95 | 45.65 | 95.93 |
| **ProCHO 5** | 24.80 | 49.52 | 94.73 |
| **PowerCHO 2** | 34.71 | 62.93 | 95.47 |
| **EX-Cell Advanced** | 35.12 | 66.86 | 94.94 |
| **OptiCHO** | 34.10 | 46.04 | 94.21 |
| **1×10^6^ cells/mL Inoculation Density** | | | |
| **Dynamis** | 52.60 | 54.91 | 95.93 |
| **ProCHO 5** | 27.52 | 52.87 | 94.73 |
| **PowerCHO 2** | 35.56 | 74.01 | 95.47 |
| **EX-Cell Advanced** | 36.45 | 69.85 | 94.94 |
| **OptiCHO** | 41.90 | 53.69 | 94.21 |
