# Supplementary material for: Impact of media and antifoam selection on monoclonal antibody production and quality using a high throughput micro‐bioreactor system
Source: Biotechnol Prog. 2017 Nov 16;34(1):262–70. doi: 10.1002/btpr.2575 (PMC5821576; doi:10.1002/btpr.2575)
Supplement: Supplementary file 6 — Supplementary Table 2 [file BTPR-34-262-s006.docx]

| **Media** | **Antifoam** | **Antifoam Added**  **(ppm)** | **Mean IVCD**  **(10^6^ cells-d/mL)** | **Titer**  **(mg/L)** | **Q_p_**  **(pg/cell-d)** |
| --- | --- | --- | --- | --- | --- |
| **ProCHO 5** | **204** | 90 | 0.54 | - | - |
|  | **C** | 60 | 18.56 | 39.84 | 2.28 |
|  | **EX-Cell** | 11 | 15.17 | 28.07 | 1.92 |
|  | **SE-15** | 60 | 17.51 | 28.28 | 1.62 |
|  | **Y-30** | 90 | 5.74 | - | - |
| **PowerCHO 2** | **204** | 90 | 1.76 | - | - |
|  | **C** | 60 | 34.97 | 61.62 | 1.75 |
|  | **EX-Cell** | 11 | 28.60 | 55.86 | 2.00 |
|  | **SE-15** | 60 | 29.00 | 50.74 | 1.78 |
|  | **Y-30** | 90 | 30.90 | 45.37 | 1.47 |
| **EX-Cell Advanced** | **204** | 90 | 0.78 | - | - |
|  | **C** | 60 | 23.40 | 46.50 | 2.25 |
|  | **EX-Cell** | 11 | 30.48 | 56.50 | 1.89 |
|  | **SE-15** | 60 | 27.00 | 48.07 | 1.76 |
|  | **Y-30** | 90 | 0.99 | - | - |
